# Ribosomal accretion, apriorism and the phylogenetic method: a response to Petrov and Williams

**DOI:** 10.3389/fgene.2015.00194

**Published:** 2015-06-02

**Authors:** Derek Caetano-Anollés, Gustavo Caetano-Anollés

**Affiliations:** ^1^Carl R. Woese Institute for Genomic Biology, University of Illinois, Urbana, IL, USA; ^2^Evolutionary Bioinformatics Laboratory, Department of Crop Sciences, University of Illinois, Urbana, IL, USA

**Keywords:** coaxial helical stacking, molecular evolution, ribosome origins, rRNA structure

## Abstract

Historical (ideographic) and non-historical (nomothetic) studies of ribosomal accretion appear to arrive at diametrically opposite conclusions. Phylogenetic analysis of thousands of RNA molecules and protein structures in hundreds of genomes supports the structural origin of the ribosome in RNA decoding and ribosomal mechanics. Predictions from extant features in a handful of rRNA structural models of the large ribosomal subunit support its origin in protein biosynthesis. In recent correspondence, one of us reported that correcting dismissals of conflicting data and avoiding unwarranted assumptions of the nomothetic method reconciled conclusions. In response, Petrov and Williams dismissed our arguments claiming we did not understand their algorithmic model of ribosomal apical growth. Instead, they controverted the historical approach. Here we show that their objections to the phylogenetic method are unjustified, that their algorithm subjectively guarantees back-in-time molecular deconstructions toward the protein biosynthetic core, and that processes of ribosomal growth are much more complex. We prompt abandoning apriorism, decreasing *ad hoc* hypotheses and integrating historical and non-historical scientific methods.

## “Eppur si Muove”

In recent correspondence, one of us challenged methods and conclusions supporting the claim that the large subunit of the ribosome originated in rRNA structures responsible for protein biosynthesis (Caetano-Anollés, 2015). The study was based on the identification of insertions of “branch” helices onto preexisting coaxially stacked “trunk” helices in a handful of crystallographic models (Petrov et al., 2014). This information was then used to build a model of growth that added concentric layers around structures of the peptidyl transferase center (PTC), which were considered the origin and most ancient “heart” of the molecule. This “onion” model ordered “ancestral expansion segments” (AES) of rRNA in time using universal statements of ribosomal accretion: (i) “an helix will appear before an adenosine stack in an A-minor motif of rRNA” (Bokov and Steinberg, 2009), and (ii) “a coaxially stacked helical trunk will appear before its inserted branch” (Petrov et al., 2014). Caetano-Anollés (2015) found that the study, which embodied a nomothetic (non-historical, universal, predictive) approach to science, dismissed conflicting “branch-to-trunk” directionalities, produced ambiguous outcomes, and was supported by unwarranted assumptions. In contrast, a recent ideographic (historical, retrodictive) approach reconstructed phylogenetic trees of ribosomal structural components from shared and derived features of rRNA structure (Harish and Caetano-Anollés, 2012). These trees represented falsifiable historical models of the origin and evolution of the ribosome. Ideographic approaches such as these search for “discovery operations,” sets of decision rules used to select empirical tests capable of decisive falsification of competing explanatory hypotheses (Grant, 2002). They do not commit to general principles of process (Laplacian “demons”; Sober and Steel, 2014) as nomothetic methods do. Instead, discovery operations generally take the form of mutual optimizations of models of change, statements of history (trees with and without reticulations), and data that is historically useful (Grant and Kluge, 2009). Since correcting dismissals of conflicting data and avoiding unwarranted assumptions appeared to reconcile conclusions of the nomothetic and ideographic studies, Caetano-Anollés (2015) prompted testing the predictive utility of nomothetic models with phylogenetic methods. His hope was to gain insight into putative “molecular fossils” and the epistemic relation that connects the present to the past (Sober and Steel, 2014).

In response, Petrov and Williams (2015) disparaged the concerns raised by Caetano-Anollés (2015) about their “insertion fingerprints.” Instead, the authors opted to controvert the phylogenetic method because it arrived to an opposite conclusion. Their position was one of confirmation: “*There is broad consensus … that the ribosome is the only source of defined-sequence protein in extant and ancestral biological systems … that the lineage of the translation system maps out the canonical tree of life… that the catalytic peptidyl transferase center is the oldest part of the large ribosomal subunit*.” However, there is little corroboration content in grand hypotheses that have not yet endured severity of test, such as the rooting of the tree of life, the origin of the catalyzed peptide bond, or the structural origin of the ribosome [see Lienau and DeSalle (2009, 2010), for discussions]. In defense of their nomothetic method, they treated the phylogenetic approach exemplified by the work of Harish and Caetano-Anollés (2012) disdainfully using the Garbage-in Garbage-out (GiGo) computing adagio, implying that the parsimony method of phylogenetic systematics does not follow the principle of evidence, content and degree of corroboration. Such statements are misleading and require a response. Parsimony and the phylogenetic method remain widely used and powerful approaches since they were introduced over four decades ago (Grant and Kluge, 2009).

Given the tone of the correspondence, it may seem unproductive to continue a conversation that would attempt to reconcile the nomothetic views of an apparent majority with the benefits of phylogenetic retrodiction for the sake of unraveling ribosomal origins. After all, Petrov and Williams appear the self-proclaimed holders of the ideographic truth: “*one sees little gain in testing a theory that is generally accepted and well supported by a broad variety of other data*.” We therefore veer the conversation toward the issue of ribosomal growth that triggered the debate in the first place, providing some few clarifications about the phylogenetic method.

## Apical versus Basipetal Ribosomal Growth

**FIGURE 1:**
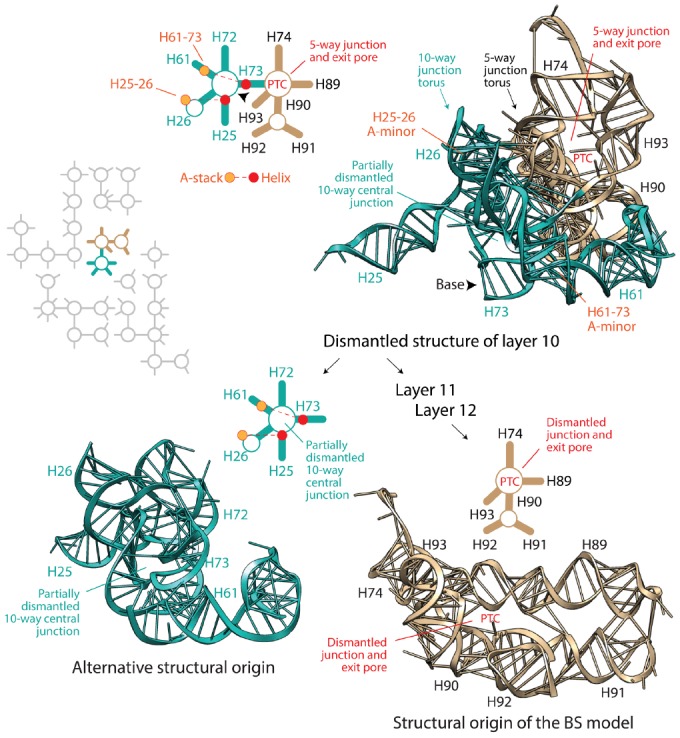
**Which dismantled structure is core?** The BS algorithm dismantles the large subunit rRNA molecule in layers, working backward in time, from the most peripheral components (layer 1) toward the central peptidyl transferase canter (PTC). The diagram shows the dismantled structure of layer 10 and two possible points of origin derived from it (see Bokov and Steinberg, 2009). The flow diagram in the right describes the removal of substructures according to the BS model, which arrives to the tan-colored structural model of the PTC. In turn, dismantling the PTC results in one of many equally likely structural origins (not made explicit by Bokov and Steinberg, 2009), which is shown in the aqua-colored structure in the left. Structural models derived from PDB entry 3R8S are complemented with schematic representations of secondary structures; helical tracts are depicted with lines and junctions with circles. The two A-minor interactions associated with structures in layer 10 are also indicated. The arrowheads point toward the base of the dismantled molecule. The inset shows a schematic representation of layer 10 structures surrounded by dismantled rRNA structures in gray. Both alternative points of origin of large subunit rRNA are equally well packed and central. Both contain torus-like substructures that juxtapose each other, and could be origins of the ribosomal molecule.

Petrov and Williams (2015) state that the Petrov et al. (2014) model of accretion follows the footsteps of the Bokov and Steinberg (2009) model, which we abbreviate “BS,” claiming that their model “*has subsumed the Steinberg method and is dependent on it*.” The BS model uses inductive reasoning to “polarize” molecular growth with the “old helix-new stack” scheme of A-minor interactions. Argumentation is risky because the age of structures holding the helix and adenosine stack pairs could be older than the helix-stack interaction, nullifying the inductive argument and the predictive algorithmic methodology of ribosomal dismantling it supports. However, the phylogenetic method that Petrov and Williams disparages confirms that in ∼80% of cases, the helix is older than the stack (Harish and Caetano-Anollés, 2012). Thus, nomothetic-ideographic reconciliation can be useful. We note however that nomothetic statements of universality can be violated. Reconciling the relative age of interacting A-minor components with ages derived from phylogenetics does not eliminate the possibility that the interaction was established well after the two supporting structures appeared in evolution. Besides the “old helix-new stack” scheme, the BS model also assumes that the ribosome must be dismantled back in time by eliminating the most terminal pieces. This hypothesis can be rephrased as an “apical” model of growth in which new branches always grow from old trunks, as opposed to a “basipetal” model of growth, in which branches must be older. Apical growth results in layering of structures (not necessarily in 3-dimensions); the internal layers are older that the external ones. The BS model applies this second universal principle (reductively) to systematically dismantle (back in time) the modern large ribosomal subunit, helix by helix, from the periphery to its core (i.e., basipetally), respecting the “old helix-new stack” scheme. Petrov and Williams (2015) state: “*the PTC origin of the ribosome, contrary to the claim of the author, was not an a priori hypothesis of Steinberg, but was one of the primary results of the method*.” However, the algorithmic implementation of ribosomal dismantling unfolds naturally from the periphery toward the large basal junctions of the molecule as the algorithm travels back in time. Since most helices of A-minor motifs are concentrated in the PTC region, dismantling even guarantees *a priori* convergence toward the PTC heart. Moreover, algorithmic steps become subjective once few structural layers are left to dismantle. In fact, the last three layers depend on only two A-minor motifs, which allow for several remaining structures of the central 10-way junction or the PTC to become the origin of the molecule. However, the BS model chooses subjectively always the PTC structures (Figure 1). Even in its last step, helix H73, which is basal to the molecule and connects the PTC to the central 10-way junction of the large subunit, is eliminated (violating the apical growth model), even though H73 could have been the origin of the molecule. Remarkably, removal of the assumption of universal apical growth allows for peripheral structures supporting ribosomal mechanics of the central, L1 and L7-12 protuberances to become points of ribosomal origin without violating the “old helix-new stack” scheme of the dismantling algorithm (Harish and Caetano-Anollés, 2012; Caetano-Anollés, unpublished). From an engineering point of view this makes sense. Some of these structures (e.g., AES1-39; Réblová et al., 2012) are central moving pieces of the ribosomal “turnstile” (Achenbach and Nierhaus, 2015): once created they must be pushed outward for them to operate.

**FIGURE 2:**
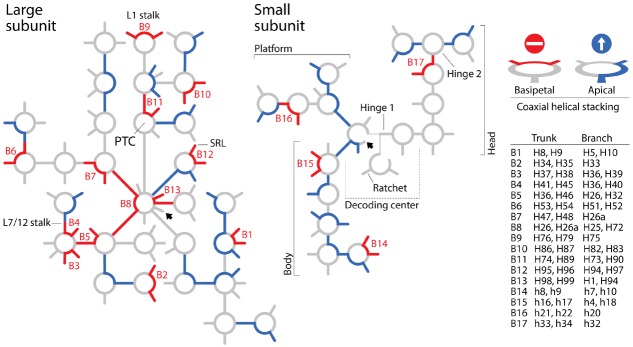
**Roadblocks to apical growth.** Patterns of coaxial helical stacking in rRNA define “branch-to-trunk” directionalities that violate the “onion” apical growth model of Bokov and Steinberg (2009) and Petrov et al. (2014). Secondary structure models of the small and large subunits of the *Escherichia coli* ribosome are depicted with schematic representations of helical tracts (lines) and junctions (circles). Unambiguous coaxial helical stacking regions in three-way and higher order junctions associated with putative sites of insertions (Petrov et al., 2014) were indexed by examining structural models and confirmed by consulting descriptions of others (e.g., Laing and Schlick, 2009; Laing et al., 2009). Coaxial helical stacking regions exhibiting basipetal and apical branch-to-trunk insertion directionalities are colored red (roadblocks) and blue (throughfares), respectively. When traveling from the base (arrowhead) to the periphery of the molecules, basipetal directionalities violate the “onion” model and defeat the BS model that supports nomothetic studies of ribosomal evolution. Stacked helices in B3, B4, and B9 subtend fundamental structures supporting translocation mechanics of the large subunit and stacked helices B11 subtend half of the PTC. Patterns of coaxial helical stacking falsify an origin of the ribosome in the PTC (discussed in Caetano-Anollés, 2015). SRL, alpha-sarcin-ricin loop.

To summarize, the BS model of apical growth establishes *a priori* an origin of the large subunit molecule in the PTC through its algorithmic implementation. It is likely however that Petrov and Williams will claim that the dismantling algorithm must converge to functional centers. This of course would negate the possibility of co-option, which pervades biology (e.g., metabolism; Teichmann et al., 2001), and has been used to explain ribosomal origins (Harish and Caetano-Anollés, 2012). Similarly, it is likely that they will see no harm in disregarding branch-to-trunk directionalities contradicting the BS model. In their response, they divert attention from the subject by introducing the concept of “trunk-branch polymorphism”: “*a branch helix can be inserted into a trunk helix (into a stem) forming a Y, or into a loop, capping the helix and forming a T*.” However, Petrov et al. (2014) never mentioned in their paper the existence of such polymorphisms, their possible effects on definitions of trunks and branches, and any possible impact of helix capping and fraying (Lee and Guttell, 2014) on insertion fingerprints. Moreover, polymorphisms appear to describe the place of original insertion and not its directionality. Consequently, there is still no justification for purposely flipping branch-to-trunk directionalities of the T-shaped AES1-39 junction (compare Table S3 of Petrov et al., 2014 with Figure 1 of Caetano-Anollés, 2015) and of AES14-16 and AES22-23, other than shifting the origin of the ribosome from structures supporting ribosomal mechanics to the PTC. In fact, hidden in supporting information we find that the chronology of ribosomal dismantling forces “*machinery required for translocation “to appear” at some stages of ribosomal development*” (Petrov et al., 2014). If additional *ad hoc* hypotheses of structural layering (“onions”), A-minor interactions (absent in AES1-39), exclusion of mechanical structures, or trunk-branch polymorphism are used to support flipping and avoid violation of the apical growth model, then the branch-to-trunk predictive scheme is defeated. Increasing the number *ad hoc* hypotheses is inversely proportional to content of corroboration (Grant and Kluge, 2009). It increases apriorism. More seriously, each departure from the apical model requires individual mechanistic and predictive justification. There are 13 putative insertions in branching junctions of the large subunit alone that violate the BS model (B1-B13), including AES1-3 (which has not been flipped) at the heart of the PTC! All of these basipetal insertions are *explananda* that *must* be explained since they refute the “onion” model of growth (Figure 2). In contrast with the analysis of A-minor interactions, phylogenetic assignments of branch-trunk ages falsify half of the remaining 12 apical insertions that are compatible with the BS model (Caetano-Anollés and Caetano-Anollés, manuscript submitted). This shows that the ribosomal core grows through processes that are much more complex than those driving the simplistic BS model.

## Apriorism and the Phylogenetic Method

Despite of its usefulness, Petrov and Williams (2015) controvert the phylogenetic method listing technical objections that are misleading or inexistent. First, they are incorrect when mentioning that phylogenetic analysis of rRNA uses “*cartoon-level*” secondary structure information “*without incorporating information from three dimensional structure*.” Phylogenetic alignments of rRNA incorporate both sequence and structural information present in thousands of molecules from organisms representing the three domains of life (see details in Methods and S1 text; Harish and Caetano-Anollés, 2012). Alignments are guided by information present in both (i) high-resolution 3D structural models, and (ii) secondary structure models inferred by covariation-based comparative sequence analysis (with prediction accuracies of up to 96%, Gutell et al., 2002), which were manually and carefully annotated during more than a decade. Sequence and structural information was then encoded in DCSE format using the MARTEN module of NOBAI (Knudsen and Caetano-Anollés, 2008), and the resulting phylogenetic matrices used in tree reconstruction. Second, rRNA structures are not folds inferred with the Vienna RNA package as Petrov and Williams claim. Instead, rRNA sequence and structural alignments are used to define structural homologies by topographic correspondence, with substructures being mapped in space in the context of the entire molecule and then tested to determine if they represent true homologies acquired from a common ancestor (Harish and Caetano-Anollés, 2012). Histories and lineages are not “*based on statistics from secondary structure prediction of local RNA elements that are extracted from large RNAs*”. “*Output from Vienna*” is not used to build “*phylogenetic trees of rRNA fragments*.” If Petrov and Williams cared to study the phylogenetic methodology, they would have realized that Harish and Caetano-Anollés (2012) analyze encodings from molecular morphometrics and not molecular mechanics. Also, they would have appreciated that both molecular morphometrics and molecular mechanics of RNA carry congruent and considerable evolutionary signal (Caetano-Anollés, 2005; Sun and Caetano-Anollés, 2007). Third, the ribosomal accretion process implies a general tendency toward molecular growth, which is necessarily linked to a tendency toward conformational order. The phylogenetic method makes use of this tendency to incorporate the “arrow of time,” enabling the study of ribosomal origin. Accretion embodies growth by addition of individual nucleotides or pairs of them in helices, or by insertion of larger fragments. In fact, Petrov et al. (2014) measured the size of the large subunit of rRNA and revealed a tendency of growth in eukaryotic molecules. These growth tendencies inspire the BS model and define AES. These same tendencies enhance the chances of establishing intramolecular interactions that are stabilizing, the existence of which Petrov and Williams list in detail (base stacking, A-minor interactions, tetraloops, etc). Therefore, it is not surprising that these growing interactions “lock-in” RNA molecules into few conformations in molecular evolution. Finally, the six technical objections they specifically raise are based on false premises:

(1) Surviving molecular progeny do not have to be more or less “*stable than their ancestors*” in evolution. Accretion implies ensembles of structural modules of different age, both in the BS and phylogenetic models, with properties of molecular flexibility and robustness distributing along the nested lineages of the tree of life (e.g., Caetano-Anollés, 2002, 2005; Sun et al., 2010).
(2) The claim that “thermodynamic stabilities and/or conformational entropies of rRNA elements change systematically over time at a rate that is uniform over the population of rRNA elements” is also incorrect and is not an assumption of the phylogenetic method. Polarization of character states transformation using a biophysical rationale is applied a posteriori to tree optimization for the sole purpose of rooting the trees. These transformations do not force uniform rates of change or prohibit the evolutionary appearance of short or long structures along any molecular lineage as Petrov and Williams affirm. Instead, optimization of character change in the rRNA molecules of different organisms defines the topology of the unrooted trees, which are then rooted by attaching a hypothetical ancestor to the branch that yields minimum increase in tree length. Thus, the long GC-rich ES27 helix in eukaryotic lineages can appear as a derived structure in reconstructions of these types despite of its length and is not pushed to the base of the trees (more below). Remarkably, polarizing characters in the opposite direction always results in trees that are less parsimonious (Caetano-Anollés, 2005; many subsequent publications). This fact alone supports both phylogenetic character argumentation and the evolutionary tendency toward increased conformational stability of molecules.
(3) Despite Petrov and Williams’ contention that familial relationships between rRNA substructures cannot exist, accretion implies an evolutionary relationship of structural parts in lineages. Parts of molecules have history very much as molecular wholes have their own (see S1 text, Harish and Caetano-Anollés, 2012). Both the predictive BS and phylogenetic methods are driven by familial relationships of structures (branching from trunks), one physical (molecular branch outgrowths) and the other abstract (discovery operations). Their statement that “*a few short primordial RNA sequences are ancestral to other sequence elements*” contradicts their claim that the phylogenetic method forces longer molecules to become old.
(4) The study of ribosomal accretion necessitates definition of the structural modules that are being accreted. This requires exploratory “*slicing and dicing*” of molecules without compromising the historical information they hold. The phylogenetic model relies on helical segments (Bailor et al., 2010). The BS model relies on AES. Combining or revising structural modules simply changes the definition of taxa and does not invalidate phylogenetic statements (e.g., splitting helices H41–H42 still places split components at the base of the tree), which are permanently revised to increase content of corroboration. In contrast, the effect of combining or revising AES must still be evaluated for possible shifts of timeframes of accretion. We note that identification of modules is linked to establishment of homologies. In the absence of topographic correspondences, definition of homologies often requires dynamic homology analysis (Grant and Kluge, 2009) or use of hidden Markov models (Yang, 1995). Phylogenetic methods systematize homology testing. In turn, nomothetic methods cannot distinguish between ancient ancestors and ancient relatives. They cannot discern similarity due to common ancestry from similarities due to other causes, making AES a misnomer.
(5) Thermodynamic statistical parameters modeled with Vienna (e.g., Shannon entropy of the base pairing probability matrix; Knudsen and Caetano-Anollés, 2008) have not been reported for rRNA. However, they hold significant phylogenetic signal (e.g., Poczai et al., 2015). Making long lists of molecular interactions of tertiary structure that could affect phylogeny does not controvert the current use of sequences and structures or their impact in unraveling history. The phylogenetic method embraces endurance of severity of test with each new phylogenetic data set.
(6) There is no circular argument (or GiGo) in the phylogenetic method. Phylogenetic trees and data are optimized without forcing any “*theoretical approach*.” A “*path of evolution*” does not predetermine the unrooted trees that are built. RNA helices can grow or become shorter as changes distribute in trees. Figure S8 of Harish and Caetano-Anollés (2012) shows for example the tracing of character state changes (molecular growth and contractions) in a tree of RNA helical substructures. The figure shows that the length of helical segments increases and decreases throughout evolutionary history and that retrodictions are not predetermined nor affected by “long branch attraction” artifacts. In contrast, the evolutionary path is predetermined by the algorithmic methods that Petrov and Williams defend. Their methods are designed *a priori* to fulfill the “onion” model of molecular growth.

## Conclusion

Nomothetic methods search for history with a Laplacian “*une intelligence*” that assumes there is a one-to-one mapping between the states of a biological system in the present and in the past (Sober and Steel, 2014). If one-to-one mappings fail, the nomothetic method fails. In turn, the phylogenetic method is built on the principle of evidence, content and degree of corroboration, which is enhanced by reciprocal illumination (Grant and Kluge, 2009). Here, the evolutionary study of ribosomal accretion makes explicit the difference between scientific methods. Petrov and Williams are optimistic and assume a Laplacian demon exists in living molecular fossils. Our analysis finds their optimism is unwarranted. While nomothetic and phylogenetic methods can complement each other if apriorism is put aside, a new integrated ideographic framework must be promoted that cares about the evolutionary effects of time.

### Conflict of Interest Statement

The authors declare that the research was conducted in the absence of any commercial or financial relationships that could be construed as a potential conflict of interest.
